# Physical activity, cognitive decline, and risk of dementia: 28 year follow-up of Whitehall II cohort study

**DOI:** 10.1136/bmj.j2709

**Published:** 2017-06-22

**Authors:** Séverine Sabia, Aline Dugravot, Jean-François Dartigues, Jessica Abell, Alexis Elbaz, Mika Kivimäki, Archana Singh-Manoux

**Affiliations:** 1 Centre for Research in Epidemiology and Population Health, INSERM U1018, Université Paris-Saclay, Hôpital Paul Brousse, Paris, France; 2 Department of Epidemiology and Public Health, University College London, London, UK; 3 INSERM U1219, University of Bordeaux, Bordeaux, France

## Abstract

**Objective:**

To test the hypotheses that physical activity in midlife is not associated with a reduced risk of dementia and that the preclinical phase of dementia is characterised by a decline in physical activity.

**Design:**

Prospective cohort study with a mean follow-up of 27 years.

**Setting:**

Civil service departments in London (Whitehall II study).

**Participants:**

10 308 participants aged 35-55 years at study inception (1985-88). Exposures included time spent in mild, moderate to vigorous, and total physical activity assessed seven times between 1985 and 2013 and categorised as “recommended” if duration of moderate to vigorous physical activity was 2.5 hours/week or more.

**Main outcome measures:**

A battery of cognitive tests was administered up to four times from 1997 to 2013, and incident dementia cases (n=329) were identified through linkage to hospital, mental health services, and mortality registers until 2015.

**Results:**

Mixed effects models showed no association between physical activity and subsequent 15 year cognitive decline. Similarly, Cox regression showed no association between physical activity and risk of dementia over an average 27 year follow-up (hazard ratio in the “recommended” physical activity category 1.00, 95% confidence interval 0.80 to 1.24). For trajectories of hours/week of total, mild, and moderate to vigorous physical activity in people with dementia compared with those without dementia (all others), no differences were observed between 28 and 10 years before diagnosis of dementia. However, physical activity in people with dementia began to decline up to nine years before diagnosis (difference in moderate to vigorous physical activity −0.39 hours/week; P=0.05), and the difference became more pronounced (−1.03 hours/week; P=0.005) at diagnosis.

**Conclusion:**

This study found no evidence of a neuroprotective effect of physical activity. Previous findings showing a lower risk of dementia in physically active people may be attributable to reverse causation—that is, due to a decline in physical activity levels in the preclinical phase of dementia.

## Introduction

The World Health Organization estimated that 47 million cases dementia existed worldwide in 2015 and predicted that this may triple by 2050 owing to ageing of the population.^1^ In the absence of a cure, prevention studies are important to identify modifiable risk factors and protective factors to delay the onset or reduce the risk of dementia.^2^ Considerable research has focused on the protective role of physical activity.^3^ ^4^ ^5^ ^6^ ^7^ Several meta-analysis of observational studies suggest a protective effect of physical activity at recommended levels for cognitive decline and risk of dementia.^3^ ^4^ ^8^ ^9^ Although intervention studies are not able to study the effects of long term physical activity, the evidence from such studies is inconsistent; some studies suggest protective effects,^10^ ^11^ whereas most recent studies show no effects.^12^ ^13^ ^14^ ^15^

Dementia is a progressive disorder involving changes over a long preclinical period,^16^ which might include alterations in physical activity. Thus, studies with short follow-up times, typically less than 10 years, cannot distinguish causal effects from those due to preclinical changes (that is, reverse causation). Accordingly, the evidence for protective effects of physical activity is more consistent in studies with short rather than long follow-up times.^4^ ^8^ ^17^ ^18^ ^19^ ^20^ To uncover the role of physical activity, we used data spanning nearly three decades to investigate the association between physical activity and cognitive function at ages 50, 60, 70, and 80 years; examine the association of physical activity with subsequent 15 year cognitive decline; assess the association between midlife physical activity and the risk of dementia; and model changes in physical activity over 28 years before diagnosis of dementia by using a backwards timescale anchored to year of diagnosis. We also examined whether associations were similar by assessing mild, moderate to vigorous, and total physical activity.

## Methods

### Population

The Whitehall II study is an ongoing cohort study of men and women originally employed by the British civil service in London based offices.^21^ A total of 10 308 people (6895 men and 3413 women, aged 35-55) were recruited over the years 1985 to 1988. Since baseline, follow-up clinical examinations have taken place approximately every five years, with the latest one completed in 2012-13.

### Patient involvement

Participants in the Whitehall II study were not involved in setting the research question or the outcome measures, nor were they involved in developing plans for recruitment, design, or implementation of the study. No participants were asked to advise on interpretation or writing up of results. However, all results are disseminated to study participants via newsletters and our website, which has a participant portal (https://www.ucl.ac.uk/whitehallII/participants/ ). Starting in 2015, a randomly selected set of participants are also involved in a consultation exercise to shape our research on ageing.

### Measures

#### Physical activity

Physical activity was assessed using a questionnaire, seven times over 28 years. At the first three assessments (1985-88, 1988-90, and 1991-93), participants were asked about the frequency and duration of participation in mildly energetic (eg, weeding, general housework, bicycle repair), moderately energetic (eg, dancing, cycling, leisurely swimming), and vigorous physical activity (eg, running, hard swimming, playing squash). Examples for each level of physical activity were provided to ease interpretation. The questionnaire was modified to reflect the Minnesota leisure-time physical activity questionnaire,^22^ starting from the 1997-99 wave, to include 20 items on frequency and duration of various activities (eg, walking, cycling, sports). For each activity, including the open ended items, we assigned a metabolic equivalent (MET) value by using a compendium of activity energy costs.^23^ One MET reflects the intensity of activity relative to lying quietly: we coded activities with MET less than 3 as mild physical activity and those with MET of 3 or more as moderate to vigorous physical activity.^24^ The sum of all physical activity denoted total physical activity. We defined “recommended physical activity” by using WHO criteria of moderate to vigorous physical activity at least 2.5 hours/week.^25^ These measures of physical activity have been shown to be associated with cardiometabolic outcomes in the Whitehall II study.^26^ ^27^ ^28^ ^29^ ^30^

#### Cognitive function

The cognitive test battery, introduced to the study in 1997-99 and repeated using the same tests at all subsequent assessments, allowed analysis using four waves of data between 1997 (age of participants 45-69 years) and 2013 (age 60-84 years).^31^ The tests have good test-retest reliability (range 0.6-0.9), assessed in 556 participants and retested within three months in 1997-99. The cognitive domains assessed were as follows. Memory was assessed using a 20 word free recall test. Participants were presented a list of one or two syllable words at two second intervals and were then asked to recall in writing as many of the words as possible in any order with two minutes to do so. Executive function was assessed with the Alice Heim 4-I test, which is composed of a series of 65 verbal and mathematical reasoning items of increasing difficulty.^32^ It tests inductive reasoning, measuring the ability to identify patterns and infer principles and rules. Participants had 10 minutes to do this section. Fluency was assessed using measures of phonemic and semantic fluency.^33^ Participants were asked to recall in writing as many words beginning with “s” (phonemic fluency) and as many animal names (semantic fluency) as they could. One minute was allowed for each test.

In addition to three cognitive domains, we created a global cognitive score incorporating all tests described above by firstly using the distribution of the first wave of cognitive data (1997-99) to standardise the raw scores for each domain to z scores (mean=0; standard deviation=1). We summed these z scores and restandardised them to yield the global score, an approach that minimises measurement error inherent in individual tests.^34^

#### Dementia

We used comprehensive tracing of electronic health records for ascertainment of dementia in three databases: the national Hospital Episode Statistics database, the Mental Health Services Data Set (MHSDS), and the mortality register. Record linkage until 31 March 2015, using ICD-10 (international classification of diseases 10th edition) codes F00, F01, F02, F03, F05.1, G30, G31.0, G31.1, and G31.8 identified cases of dementia. The NHS in the UK (England, Scotland, and Wales) provides most of the healthcare, including outpatient and inpatient care; private medical insurance, held by around 12% of the UK population (1997 figures),^35^ is mainly used for elective surgery rather than chronic conditions such as dementia. MHSDS is a national database that contains information for people in contact with mental health services in hospitals, outpatient clinics, and the community. Mortality data came from the British national mortality register (NHS Central Registry). The tracing exercise used the unique NHS identification number given to each resident in the UK. We set date of dementia at the first record of a diagnosis of dementia in any of the three databases used for ascertainment.

The validity of dementia cases in our study is supported by modelling changes in the global cognitive score over 18 years before diagnosis of dementia. These results show accelerated decline in global cognitive score in the 8-10 years before diagnosis of dementia (supplementary figure A), as has been shown in studies that use a “gold standard” dementia ascertainment procedure.^36^

#### Covariates (1985 to 2013)

Sociodemographic variables included age, sex, ethnicity (white versus non-white), marital status (married/cohabiting versus others), socioeconomic status using occupational position (three categories: high, intermediate, and low representing income and status at work), and education (five categories: less than primary school (up to age 11), lower secondary school (up to age 16), higher secondary school (up to age 18), university, and higher university degree). Occupational position and marital status were updated at each wave.

Health behaviours were assessed by questionnaire and included smoking (current smokers, ex-smokers, and never smokers), alcohol consumption (number of alcoholic drinks consumed in the previous seven days, converted to units of alcohol consumed in a week and categorized as “no/occasional alcohol consumption,” “moderate alcohol consumption” (1-14 units/week in women, 1-21 units/week in men), and “heavy alcohol consumption” (≥14 units in women, ≥21 units in men)),^37^ and dietary behaviour (frequency of fruit and vegetables consumption in a week).

Health related covariates included symptoms of anxiety and depression (30 item General Health Questionnaire),^38^ hypertension (systolic/diastolic blood pressure ≥140/90 mm Hg or use of antihypertensive drugs), prevalent diabetes mellitus (determined by fasting glucose ≥7.0 mmol/L, reported diabetes diagnosed by a doctor, or use of diabetes drugs), body mass index assessed at the clinical examination (categorised as <20, 20-25, 25-30, and ≥30), cardiovascular diseases (including coronary heart disease and stroke (identified using linkage to national hospital records)), self reported use of drugs for cardiovascular disease, and the physical component score of the SF-36 (self rated physical functioning).^39^

### Statistical analysis

We did three sets of analyses, described below (see supplementary figure B for flowchart). For the first two analyses, we categorised physical activity (total, mild, and moderate to vigorous) into approximate thirds; we also categorised physical activity at moderate to vigorous intensity by using the WHO recommended level of at least 2.5 hours/week. For the final analysis, we modelled physical activity (total, mild, and moderate to vigorous) as a continuous variable (hours/week). When cognitive function is the outcome, we present results using the global cognitive score; results using individual cognitive tests are shown in the supplementary materials.

#### Association between physical activity and cognitive function

These analyses were based on 7424 participants with data on cognitive function (added to the study protocol only in 1997-99). We first examined whether the association between physical activity over the follow-up (from 1997-99 to 2012-13) and cognitive function (time varying from 1997-99 onwards) changed with age by using linear mixed effect models with age as timescale and time varying physical activity and cognitive function. This model also included age (centred at mean age (65 years) over the follow-up period), age^2^, five year birth cohort, sociodemographic factors, health behaviours, and interaction terms of age and age^2^ with both time varying physical activity categories and time invariant variables (sex, education, ethnicity); see equation 1 in supplementary methods. We then adjusted the analyses for time dependent health related measures. To ease presentation of the results, we compared estimated differences (and 95% confidence intervals) in cognitive scores in the intermediate and high physical activity categories against “low” physical activity at age 50, 60, 70, and 80 years. For the test of change in the association between physical activity level and cognition with age, we examined whether the interaction terms of physical activity groups with age and age^2^ improved model fit by using the Wald test.

We then examined whether physical activity in 1997-99 was associated with cognitive decline over 15 years (1997-99 to 2012-13) by using linear mixed effect models, with intercept and slope fitted as random effects.^29^ The basic model included the following terms: time of follow-up starting from 1997-99, time^2^, age in 1997-99 (centred at mean age (55 years)), sex, physical activity, their interactions with time, and the interaction between age and time^2^ to account for accelerated cognitive decline at older ages (supplementary methods, equation 2). We used the interaction between physical activity and time to test for differences in cognitive decline between the categories of physical activity. The analyses were further adjusted for sociodemographic variables, behavioural variables, and their interactions with time. Finally, we added health related variables with their interactions with time to the model. We examined whether age modified the association of physical activity with cognitive decline by using interaction terms between physical activity, time, and age (continuous variable).

#### Association between physical activity and risk of incident dementia

We examined the association of physical activity (categories and then as a continuous variable) assessed in 1985-88 (study recruitment) and dementia in all 10 308 participants recruited to the study in 1985-88 by using Cox regression with age as the timescale (supplementary methods, equation 3). Participants were censored at date of record of dementia, death (to account for competing risk of mortality), or 31 March 2015, whichever came first. Covariates (sociodemographic variables, health behaviours, health status) were drawn from the 1985-88 wave.

#### Trajectories of physical activity before dementia

The aim of these analyses was to examine differences in trajectories of physical activity (hours/week of total, mild, and moderate to vigorous activity) in people with dementia compared with those free of dementia. We used a backwards timescale,^36^ such that year=0 in the analysis was the year of dementia diagnosis for cases, year of death for those who died during the follow-up, and end of follow-up (31 March 2015) for all others. We modelled physical activity trajectories over 28 years before year 0 by using mixed effects models, with random intercept and slope, and hours/week of physical activity as the dependent variable. Cubic regression splines, used in exploratory analyses to verify the shape of trajectories of physical activity, suggested a cubic polynomial shape (supplementary figure C). Thus, we used a mixed model with dementia (coded as 1 for cases and 0 for all others) and its interaction with slope terms (time, time^2^, and time^3^) to test for differences in physical activity trajectories between dementia cases and all others by assessing whether the interaction terms improved the fit of the model with the Wald test. This method also allowed the estimation of differences in physical activity between cases and non-cases for each year over the 28 year follow-up. The analysis was adjusted for five year cohort of birth and time dependent (when relevant) sociodemographic and behavioural covariates and interactions of time invariant variables with time, time^2^, and time^3^ (supplementary methods, equation 4). We repeated these analyses using a case-control approach to better control for confounders and period effects inherent to the diagnosis of dementia (see supplementary methods).

#### Sensitivity analysis

Data on type of dementia were incomplete in the electronic health records, not allowing us to test the salience of physical activity by subtypes of dementia, such as Alzheimer’s disease. However, as complete history of cardiovascular disease in our participants was available over the follow-up, we categorised dementia cases into dementia with and without a history of cardiovascular disease (myocardial infarction or stroke). For each of these outcomes (the other being censored in the analysis at age of dementia diagnosis), we used Cox regression to examine associations with physical activity. Trajectories of physical activity before dementia using a three category dementia variable (no dementia, dementia with cardiovascular disease history, and without cardiovascular disease history) allowed us to examine changes in physical activity in relation to these subtypes of dementia.

To determine whether poor sensitivity of our method of dementia ascertainment affected the results, we simulated scenarios with differential misclassification—that is, the hypothesis that the probability of dementia misclassification depends on physical activity—by using an SAS macro provided by Fox et al.^40^ We allowed the sensitivity to vary between 50% and 80% and specificity between 97% and 100% with a trapezoidal probability density function. We simulated two scenarios: one in which the correlation between the sensitivity distribution in people following recommendations and the sensitivity distribution in all others was 0.8 and then one in which it was 0.6 (and similarly for the correlations of the specificity distributions); note that a correlation of 1 corresponds to non-differential misclassification.

## Results

Over a mean follow-up of 26.6 (SD 4.5; range 0.18-29.6) years, a total of 329 cases of dementia were recorded. The mean age at diagnosis was 75.0 (SD 5.4; interquartile range 72.0-79.2; range 53.4-83.6) years, and 73% (n=240) of the cases were recorded in the last five years of follow-up. Increasing age (hazard ratio per 1 year greater age at study baseline 1.21, 95% confidence interval 1.19 to 1.24), female sex (1.58, 1.27 to 1.96), and education less than secondary school diploma (1.76, 1.41 to 2.19) were associated with greater hazard of dementia. Table 1 shows characteristics of the study population (n=10 308).

**Table 1:** Baseline characteristics of study population (n=10 308)*. Values are numbers (percentages) unless stated otherwise

Characteristic	Dementia status at end of follow-up		P value		Following recommendations of 2.5 hours/week of moderate to vigorous physical activity		P value
	No dementia (n=9979)	Dementia (n=329)			Yes (n=5347)	No (n=4961)	
Mean (SD) age, years	44.8 (6.0)	50.5 (4.7)	<0.001		44.5 (6.0)	45.4 (6.1)	<0.001
Women	3269 (32.8)	144 (4448)	<0.001		1278 (23.9)	2135 (43.0)	<0.001
Non-white	1076 (10.8)	51 (16)	0.007		404 (7.6)	723 (14.6)	<0.001
University degree or higher	2601 (26.1)	63 (19)	0.005		1461 (27.3)	1203 (24.2)	<0.001
Low occupational position	2206 (22.1)	131 (40)	<0.001		816 (15.3)	1521 (30.7)	<0.001
Married/cohabiting	7373 (73.9)	235 (71)	0.32		4175 (78.1)	3433 (69.2)	<0.001
Current smokers	1823 (18.3)	68 (21)	0.27		906 (16.9)	985 (19.9)	<0.001
Heavy alcohol consumption†	1707 (17.1)	44 (13)	0.08		1003 (18.8)	748 (15.1)	<0.001
Poor diet‡	4170 (41.8)	135 (41)	0.85		2130 (39.8)	2175 (43.8)	<0.001
Mean (SD) total physical activity, hours/week	11.0 (8.8)	10.7 (8.3)	0.28		14.4 (9.1)	7.2 (6.6)	<0.001
Mean (SD) mild physical activity, hours/week	7.2 (6.9)	7.4 (7.0)	0.60		8.1 (7.2)	6.3 (6.6)	<0.001
Mean (SD) moderate to vigorous physical activity, hours/week	3.8 (4.2)	3.3 (3.7)	0.04		6.3 (4.5)	1.0 (0.8)	<0.001
Moderate to vigorous physical activity ≥2.5 hours/week	5190 (52.0)	157 (48)	0.13		5347 (100)	0 (0)	–
Diabetes	90 (0.9)	10 (3)	<0.001		37 (0.7)	63 (1.3)	0.003
Mean (SD) body mass index	24.6 (3.5)	25.7 (4.0)	<0.001		24.5 (3.3)	24.8 (3.8)	<0.001
Hypertension	1876 (18.8)	93 (28)	<0.001		977 (18.3)	992 (20.0)	<0.001
Cardiovascular disease	116 (1.2)	6 (2)	0.28		58 (1.1)	64 (1.3)	0.33
Cardiovascular disease drugs	322 (3.2)	18 (5)	0.03		137 (2.6)	203 (4.1)	<0.001
Mean (SD) General Health Questionnaire score	3.6 (5.4)	4.0 (5.8)	0.25		3.4 (5.1)	3.9 (5.8)	<0.001

* Al data drawn from 1985-88, baseline of Whitehall II study.

^†^ Defined as ≥14 units/week in women and ≥21 units/week in men.

‡ Corresponds to fruit and vegetable consumption less than once a day.

### Association between physical activity and cognitive function

Of the 7424 participants in these analyses, 42% (n=3144) had cognitive data at all four waves, 29% (n=2168) at three waves, 15% (n=1091) at two waves, and 14% (n=1021) at only one wave. Supplementary table A shows the association between physical activity and global cognitive z score at ages 50, 60, 70, and 80 years in models adjusted for sociodemographic and behavioural factors. For all measures of physical activity, the association with the global cognitive score was stronger at older ages (all P for interaction with age <0.05). Although these differences were considerably attenuated in the fully adjusted models, the association between physical activity and cognitive function was stronger at older ages except for mild physical activity (table 2). Compared with people not following physical activity recommendations, those following recommendations had similar cognitive function at age 50 (difference in global cognitive z score 0.03, 95% confidence interval −0.01 to 0.07), but at age 80 they had on average a global cognitive z score 0.12 (0.07 to 0.18) standard deviations higher. Mutual adjustment for mild and moderate to vigorous intensity physical activity showed similar findings (P for interaction with age=0.23 and 0.005, respectively; data not tabulated). The results were similar when cognitive domains were examined separately (supplementary tables B-D).

**Table 2:** Association of physical activity with performance on global cognitive z score at age 50, 60, 70, and 80 years

	Difference in cognitive function* (95% CI)				P value for interaction with age
	Age 50	Age 60	Age 70	Age 80	
**Total physical activity**					
<8 hours/week	Reference	Reference	Reference	Reference	<0.001
8-12 hours/week	0.00 (−0.04 to 0.04)	−0.02 (−0.04 to 0.01)	0.04 (0.01 to 0.07)	0.17 (0.10 to 0.25)	
≥12 hours/week	0.00 (−0.04 to 0.05)	−0.01 (−0.03 to 0.02)	0.04 (0.01 to 0.06)	0.14 (0.07 to 0.21)	
**Mild physical activity**					
<5 hours/week	Reference	Reference	Reference	Reference	0.21
5-9 hours/week	0.00 (−0.04 to 0.05)	0.00 (−0.02 to 0.02)	0.03 (0.01 to 0.06)	0.10 (0.02 to 0.18)	
≥9 hours/week	−0.01 (−0.06 to 0.04)	0.00 (−0.03 to 0.02)	0.02 (−0.01 to 0.05)	0.07 (−0.01 to 0.15)	
**Moderate to vigorous physical activity**					
<2 hours/week	Reference	Reference	Reference	Reference	0.001
2-4 hours/week	0.02 (−0.03 to 0.06)	0.01 (−0.01 to 0.03)	0.05 (0.02 to 0.07)	0.13 (0.06 to 0.20)	
≥4 hours/week	0.04 (0.00 to 0.08)	0.00 (−0.02 to 0.02)	0.04 (0.01 to 0.06)	0.14 (0.08 to 0.21)	
**Physical activity recommendations** **†**					
MVPA <2.5 hours/week	Reference	Reference	Reference	Reference	<0.001
MVPA ≥2.5 hours/week	0.03 (−0.01 to 0.07)	−0.01 (−0.03 to 0.01)	0.02 (0.00 to 0.05)	0.12 (0.07 to 0.18)	

Models adjusted for ethnicity, education, five year birth cohort, and time dependent occupational position, marital status, smoking status, alcohol consumption, fruit and vegetable consumption, hypertension, diabetes, body mass index, General Health Questionnaire score, cardiovascular disease, cardiovascular disease drugs, and SF-36 physical component score.

* Estimated differences (expressed in standard deviations using distribution of first wave of cognitive data in 1997-99) in cognitive function at age 50, 60, 70, and 80 years drawn from single longitudinal model using age as timescale.

^†^ Recommended level corresponds to moderate to vigorous physical activity (MVPA) ≥2.5 hours/week.

Mean 15 year decline in the global cognitive z score was 0.61 (0.59 to 0.63) of the baseline standard deviation. We found no association between physical activity, assessed in 1997-99, and subsequent 15 year decline in the global cognitive z score (table 3); age did not modify this association (all P for interaction >0.19). We obtained a similar pattern of results for the three cognitive domains (supplementary tables E-G).

**Table 3:** Association of physical activity in 1997-99 with cognitive decline over 15 years (1997-99 to 2012-13), using global cognitive z score

	Adjusted for age and sex			Adjusted for sociodemographic and behavioural factors*			Fully adjusted†	
	15 year cognitive decline‡ (95% CI)	P value§		15 year cognitive decline‡ (95% CI)	P value§		15 year cognitive decline‡ (95% CI)	P value§
**Total physical activity**								
<8 hours/week	−0.61 (−0.65 to −0.58)	Reference		−0.63 (−0.66 to −0.59)	Reference		−0.63 (−0.66 to −0.59)	Reference
8-12 hours/week	−0.62 (−0.65 to −0.59)	0.48		−0.62 (−0.66 to −0.59)	0.80		−0.63 (−0.66 to −0.59)	0.73
≥12 hours/week	−0.63 (−0.66 to −0.60)	0.68		−0.63 (−0.66 to −0.60)	0.94		−0.63 (−0.66 to −0.60)	0.99
**Mild physical activity**								
<5 hours/week	−0.61 (−0.65 to −0.57)	Reference		−0.62 (−0.66 to −0.59)	Reference		−0.62 (−0.66 to −0.59)	Reference
5-9 hours/week	−0.63 (−0.66 to −0.60)	0.56		−0.63 (−0.66 to −0.60)	0.73		−0.63 (−0.66 to −0.60)	0.67
≥9 hours/week	−0.62 (−0.66 to −0.59)	0.43		−0.63 (−0.66 to −0.60)	0.72		−0.63 (−0.67 to −0.60)	0.69
**Moderate to vigorous physical activity**								
<2 hours/week	−0.61 (−0.64 to −0.58)	Reference		−0.62 (−0.65 to −0.59)	Reference		−0.61 (−0.64 to −0.58)	Reference
2-4 hours/week	−0.62 (−0.66 to −0.58)	0.13		−0.63 (−0.66 to −0.59)	0.26		−0.63 (−0.66 to −0.59)	0.18
≥4 hours/week	−0.64 (−0.67 to −0.61)	0.55		−0.64 (−0.67 to −0.61)	0.67		−0.65 (−0.68 to −0.61)	0.55
**Physical activity recommendations** **¶**								
MVPA <2.5 hours/week	−0.61 (−0.64 to −0.59)	Reference		−0.62 (−0.65 to −0.60)	Reference		−0.62 (−0.65 to −0.59)	Reference
MVPA ≥2.5 hours/week	−0.63 (−0.65 to −0.60)	0.45		−0.63 (−0.66 to −0.61)	0.66		−0.63 (−0.66 to −0.61)	0.55

* Adjusted for age, sex, ethnicity, education, occupational position, marital status, smoking status, alcohol consumption, and fruit and vegetable consumption.

† Additionally adjusted for hypertension, diabetes, body mass index, General Health Questionnaire score, cardiovascular disease, cardiovascular disease drugs, and SF-36 physical component score.

‡ Estimated cognitive decline over 15 years (expressed in standard deviations using distribution of first wave of cognitive data in 1997-99) as a function of physical activity.

§ P for difference in decline in global cognitive score over 15 years by physical activity groups (drawn from test of interaction between physical activity categories and time from baseline).

¶ Recommended level corresponds to moderate to vigorous physical activity (MVPA) ≥2.5 hours/week.

### Association between physical activity and dementia

Age at diagnosis of dementia was similar in the categories used to define physical activity: 74.9, 74.9, and 75.2 years in the low, intermediate, and high total physical activity categories (P=0.90). Table 4 shows no association between physical activity, assessed in 1985-88, and incidence of dementia, with follow-up until 2015. The lack of association was observed for total physical activity and both mild and moderate to vigorous intensity activity.

**Table 4:** Association between physical activity and dementia.

Physical activity in 1985-88 (mean follow-up 26.6 years)	Cases/total	Adjusted for age and sex			Adjusted for sociodemographic and behavioural factors*			Fully adjusted†	
		Hazard ratio (95% CI)	P value		Hazard ratio (95% CI)	P value		Hazard ratio (95% CI)	P value
**Total physical activity**									
<8 hours/week	142/4285	1.00	Reference		1.00	Reference		1.00	Reference
8-12 hours/week	71/2451	0.89 (0.67 to 1.18)	0.42		0.94 (0.71 to 1.26)	0.68		0.97 (0.72 to 1.29)	0.82
≥12 hours/week	116/2572	1.03 (0.80 to 1.32)	0.82		1.06 (0.82 to 1.36)	0.67		1.05 (0.82 to 1.36)	0.68
Per 1 hour/week‡	329/10 308	1.00 (0.98 to 1.01)	0.50		0.99 (0.98 to 1.01)	0.39		0.99 (0.98 to 1.01)	0.36
**Mild physical activity**									
<5 hours/week	131/4266	1.00	Reference		1.00	Reference		1.00	Reference
5-9 hours/week	108/3211	1.11 (0.86 to 1.44)	0.40		1.20 (0.92 to 1.55)	0.18		1.21 (0.93 to 1.58)	0.15
≥9 hours/week	90/2831	1.00 (0.76 to 1.31)	0.99		0.99 (0.75 to 1.31)	0.94		0.98 (0.74 to 1.30)	0.90
Per 1 hour/week‡	329/10 308	1.00 (0.99 to 1.02)	0.96		1.00 (0.98 to 1.01)	0.73		1.00 (0.98 to 1.01)	0.69
**Moderate to vigorous physical activity**									
<2 hours/week	123/3225	1.00	Reference		1.00	Reference		1.00	Reference
2-4 hours/week	89/3078	0.88 (0.67 to 1.16)	0.36		1.03 (0.78 to 1.37)	0.83		1.01 (0.76 to 1.35)	0.94
≥4 hours/week	117/4005	0.97 (0.74 to 1.25)	0.80		1.08 (0.83 to 1.42)	0.56		1.08 (0.82 to 1.41)	0.58
Per 1 hour/week‡	329/10 308	0.99 (0.96 to 1.02)	0.56		0.99 (0.97 to 1.02)	0.66		0.99 (0.97 to 1.02)	0.66
**Physical activity recommendations** **§**									
MVPA <2.5 hours/week	172/4961	1.00	Reference		1.00	Reference		1.00	Reference
MVPA ≥2.5 hours/week	157/5347	1.00 (0.80 to 1.24)	0.98		1.08 (0.86 to 1.36)	0.49		1.07 (0.86 to 1.35)	0.54

* Adjusted for age, sex, ethnicity, education, occupational position, marital status, smoking status, alcohol consumption, and fruit and vegetable consumption.

† Additionally adjusted for hypertension, diabetes, body mass index, General Health Questionnaire score, cardiovascular disease, and cardiovascular disease drugs.

‡ Estimates are from separate model with physical activity modelled as continuous variable (hour/week).

§ Recommended level corresponds to moderate to vigorous physical activity (MVPA) ≥2.5 hours/week.

### Trajectories of physical activity before dementia

**Fig 1:**
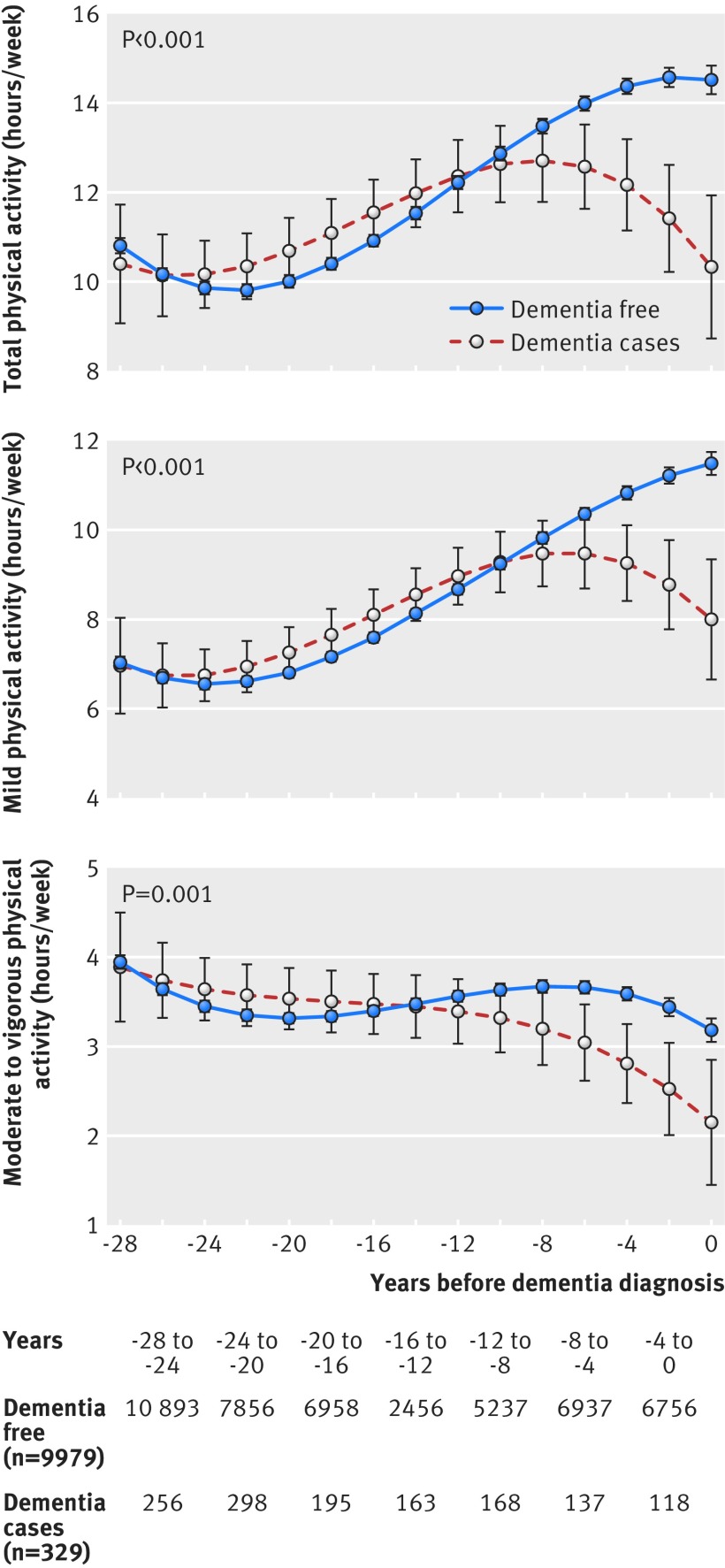
Trajectories of physical activity (hours/week) over 28 years preceding diagnosis of dementia. See accompanying data in supplementary table H

Participants following physical activity recommendations over the data waves were as follows: 52% (5347/10 308) in 1985-88, 48% (3663/7563) in 1988-90, 49% (4097/8319) in 1991-93, 51% (3623/7153) in 1997-99, 56% (3811/6786) in 2002-04, 56% (3693/6611) in 2007-09, and 55% (3402/6207) in 2012-13. Figure 1 shows that trajectories of hours/week of total, mild, and moderate to vigorous intensity physical activity differed between dementia cases and all non-cases (P≤0.001). Physical activity was lower in dementia cases than in others, starting up to nine years before diagnosis (−0.39 hour/week for moderate to vigorous intensity physical activity; P=0.05), with a difference of −4.20 hours/week (P<0.001) for total physical activity and −1.03 hours/week (P=0.005) for moderate to vigorous intensity physical activity at year 0 (the year of dementia diagnosis; supplementary table H). We found no difference in physical activity between the two groups between 28 and 10 years before diagnosis of dementia (supplementary table H). Analysis using a case-control approach showed similar results (P for difference in trajectories <0.001 for all physical activity measures; supplementary figure D and table I).

### Sensitivity analysis

We found no association between physical activity in 1985-88 and risk of dementia with (n=111) or without (n=218) a history of cardiovascular disease (supplementary tables J and K). The trajectories of physical activity over 28 years suggest that the decline in physical activity in the preclinical phase of dementia was greater in people with dementia who had a history of cardiovascular disease (supplementary figure E).

The associations between physical activity and dementia examined under the hypothesis of differential misclassification of dementia are shown in supplementary table L. They do not suggest any association between physical activity at recommended levels and risk of dementia.

## Discussion

This longitudinal study with data on physical activity spanning up to 28 years has three key findings. Firstly, the difference in the global cognitive score between people following and not following recommendations on moderate to vigorous physical activity was four times as large at age 80 as at age 50. Secondly, 15 year decline in the global cognitive score was similar in all groups defined by physical activity, irrespective of duration or intensity of activity. We found no evidence that this pattern of results varied as a function of cognitive tests. Thirdly, physical activity in midlife was not associated with risk of dementia or with dementia classified using cardiovascular history as a proxy for dementia subtypes. The findings on dementia were corroborated in analysis of trajectories of physical activity over 28 years showing that hours/week of physical activity (both mild and moderate-to-vigorous intensity) did not differ in dementia cases and non-cases between 28 to 10 years before diagnosis. However, physical activity levels began to decline in people with dementia, starting nine years before diagnosis. Taken together, these findings do not suggest a causal effect of physical activity on cognitive decline or risk of dementia. The lack of association between physical activity and cognitive decline and the divergence in physical activity trajectories emerging only in the nine years before diagnosis of dementia (that is, the preclinical phase) lead us to conclude that physical activity does not affect the risk of dementia.

### Strengths and limitations of study

This study has several strengths. Firstly, repeat assessment of physical activity over 28 years allowed us to examine trajectories of physical activity before diagnosis of dementia by using an innovative analytical strategy anchored to the cause of disease, reflected in use of the backwards timescale. Conventional analysis tends to use wave of data collection to study change, which is not optimal when preclinical disease affects risk factor levels. We were also able to assess associations with both mild and moderate to vigorous intensity physical activity. Sensitivity analysis using a case-control approach (six matched controls for each dementia case) ensured better control for confounding (by age, sex, and education) and period effects that may affect diagnosis of dementia.^36^

A key limitation of the study concerns the ascertainment of dementia based on linkage to electronic health records. In the Mayo Clinic Study of Aging and the Adult Changes study, comparison of passive case finding with an active approach showed the passive approach to have high specificity and approximately 70% sensitivity and to miss mostly milder cases of dementia.^41^ A similar pattern is likely in our study, as health coverage is universal in the UK and electronic health records have been shown to be reliable for ascertainment of dementia status.^42^ In addition, undiagnosed dementia becomes common only in very old age,^43^ and we found no evidence that physical activity at baseline affected age at diagnosis of dementia. Thus, any misclassification of dementia status is likely to be random (that is, the probability of dementia status being misclassified is independent of physical activity), and sensitivity analysis simulating differential misclassification showed our results to be robust. Furthermore, our results on cognitive decline are in accordance with other longitudinal studies showing no association between physical activity and cognitive ageing.^4^ ^8^ ^14^ Finally, another limitation concerns the change in the physical activity questionnaire in 1997-99 that could distort the trajectories of physical activity before diagnosis of dementia. However, analysis based on the case-control design, in which cases and their controls had the same questionnaires across data waves, suggested similar results.

### Comparison with other studies

Previous reviews of observational studies concluded that higher levels of physical activity are associated with a slower rate of cognitive decline.^3^ ^4^ ^8^ ^9^ These conclusions are in stark contrast to meta-analyses of intervention studies, particularly recent data, which do not show a protective effect of physical activity on cognition.^10^ ^11^ ^13^ ^14^ ^15^ This inconsistency has raised concerns about the causal nature of the association between physical activity and cognitive outcomes. Interventions use objective measures of physical activity, whereas a lot of the observational data come from self reported measures of physical activity. Our results are based on reported physical activity; however, as reporting biases at the start of the study are unlikely to be affected by dementia status (mean follow-up 26.6 years), this is an unlikely source of major bias for the observed associations between physical activity and dementia in our study. A further source of inconsistency in results in this domain is that much of the evidence comes from studies in which physical activity was measured at older age (65 years or more) and the follow-up was short (less than 10 years),^4^ ^44^ ^45^ ^46^ ^47^ making them prone to reverse causation biases. Secondly, the conclusions from some longitudinal studies do not differentiate findings on cognitive performance from those on cognitive decline.^4^ ^9^ Our analyses show that associations between physical activity and cognitive performance are stronger at older ages; a possible explanation for this pattern is decline in physical activity in people with cognitive impairment or in the preclinical phase of dementia. Thirdly, some previous studies did not include critical confounding factors, such as sociodemographic factors and health behaviours, in their analysis.^4^ ^9^

Apart from notable exceptions,^8^ ^18^ ^19^ ^48^ most previous studies on dementia are in older adults in whom preclinical dementia or cognitively impairment at recruitment into the study is difficult to rule out. Dementia is now widely recognised as involving a long preclinical period that unfolds over several years, perhaps decades,^16^ ^49^ which affects various processes, including risk factor levels. As a consequence, the risk factor-dementia association drawn from studies based on older adults is likely to be subject to reverse causation biases. In our study, physical activity started to decline up to nine years before diagnosis of dementia, resulting in lower physical activity levels in dementia cases. However, we found no differences in physical activity levels in dementia cases compared with others from year −28 to −10 before diagnosis. The results were similar for mild and moderate to vigorous intensity physical activity, suggesting that all types of physical activity decline in the years preceding dementia. Data on physical activity spanning 28 years allowed us to characterise the trajectory of physical activity and to provide potential explanations for studies in older adults with short follow-up that have found higher levels of physical activity to have a protective effect on dementia.^4^

Results from studies with a long follow-up are also inconsistent.^4^ ^8^ ^17^ ^18^ ^19^ The Caerphilly Prospective Study and the Adult Health Study found no association between midlife physical activity and incidence of dementia at older ages.^8^ ^48^ However, data from the AGES-Reykjavik Study and CAIDE (Cardiovascular Risk Factors, Ageing, and Dementia) show that people who were less active in midlife had a higher risk of dementia at older ages.^18^ ^19^ Intriguingly, the CAIDE study found no association between physical activity and dementia when analyses were based on the entire target population (n=544 (15.3%) dementia cases out of 3559 people) and electronic health records were used to identify dementia cases; in contrast, a protective effect was seen in the subsample (n=250 (16.5%) dementia cases out of 1511 participants) in which participants attended a clinical examination to have cognitive tests 20 and 28 years after recruitment to the study.^19^ Given a 1.2% higher rate of dementia when it is ascertained using a clinical examination, the reasons for this discrepancy are unclear: selective mortality and attrition may have contributed to these inconsistent results from the same study.^19^ In the Rotterdam study in adults aged 61 to 97 years at study baseline, the marginal protective effect of greater physical activity for dementia was evident only when short follow-up was used,^17^ pointing to potential biases due to reverse causation. Physical activity is known to be associated with cardiometabolic outcomes, including in our study^26^ ^27^ ^28^ ^29^ ^30^; as these are known to be risk factors for dementia,^2^ one would expect physical activity to be associated with dementia. However, as dementia is a disease of old age, participants need to live long enough to develop it. A total of 1653 participants in our study died over the follow-up; mean age at death was 67.6 years, whereas mean age at diagnosis of dementia was 75.0 years.

### Conclusion

Given rapid increases in life expectancy, dementia is increasingly a public health challenge and the need to identify modifiable risk factors that prevent or delay dementia onset has never been greater. Owing to its cardioprotective effect, physical activity has been put forward as a possible candidate. The evidence from our study, which examined both cognitive decline and dementia, challenges this hypothesis and provides no evidence for a neuroprotective effect. Physical activity levels were lower in the years leading up to diagnosis of dementia, suggesting that changes in physical activity might simply be part of the preclinical symptoms of dementia. We found no evidence of a slower rate of cognitive decline in people doing more physical activity. These findings are in accordance with results from recent randomised control trials,^10^ ^11^ ^13^ ^14^ including the most recent LIFE study based on a 24 month intervention and the Multidomain Alzheimer Preventive Trial based on a 36 month intervention, which failed to find an effect of the intervention on cognitive function and incidence of dementia.^12^ ^15^

**What is already known on this topic**

Meta-analyses of observational studies conclude that physical activity is a protective factor for cognitive decline and risk of dementia, particularly in on older adults followed for less than 10 years
However, the most recent Cochrane review on the effect of aerobic intervention on cognition did not show any evidence of benefit
The inclusion of physical activity in guidelines to tackle the burden of dementia seems not be based on robust evidence of a protective effect of physical activity

**What this study adds**

Physical activity (mild and moderate to vigorous intensity), assessed seven times over 28 years, did not have a protective effect for cognitive decline or risk of dementia
Trajectories of physical activity showed no difference between dementia cases and non-cases in the 28 to 10 years before diagnosis, with decline in physical activity starting up to nine years before diagnosis
These results suggest that a decrease in physical activity could be part of the cascade of changes occurring in the preclinical phase of dementia

## Web Extra

Extra material supplied by the author

## Notes

Contributors: ASM and SS developed the hypothesis and study design. SS and AD did the statistical analysis. SS wrote the first and successive drafts of the manuscript. All authors contributed to study concept and design, analysis and interpretation of data, and drafting or critical revision of the manuscript for important intellectual content or additionally to data acquisition. ASM and MK obtained funding for the Whitehall II study. SS, AD, and ASM had full access to the data and take responsibility for the integrity of the data and the accuracy of the data analysis. SS is the guarantor.

Ethical approval: University College London Hospital Committee on the Ethics of Human Research, reference number 85/0938. Written informed consent from participants and research ethics approvals were renewed at each contact.

Data sharing: Whitehall II data, protocols, and other metadata are available to the scientific community. Please refer to the Whitehall II data sharing policy at https://www.ucl.ac.uk/whitehallII/data-sharing.

Transparency declaration: The lead author affirms that this manuscript is an honest, accurate, and transparent account of the study being reported; that no important aspects of the study have been omitted; and that any discrepancies from the study as planned (and, if relevant, registered) have been explained.
