# Supplementary material for: Physical activity, cognitive decline, and risk of dementia: 28 year follow-up of Whitehall II cohort study
Source: BMJ. 2017 Jun 22;357:j2709. doi: 10.1136/bmj.j2709 (PMC5480222; doi:10.1136/bmj.j2709)
Supplement: Supplementary file 1 — Supplementary materials [file sabs037663.ww1.pdf]

Physical Activity, Cognitive Decline and Risk of Dementia: 28-year Follow-Up of the Whitehall II Study

Séverine Sabia, *Research Associate**[1,2]

Aline Dugravot, *Statistician*[1]

Jean-François Dartigues, *Professor*[3]

Jessica Abell, *Research Associate*[1,2]

Alexis Elbaz, *Research Professor*[1]

Mika Kivimäki, *Professor*[2]

Archana Singh-Manoux, *Research Professor*[1,2]

[1] Centre for Research in Epidemiology and Population Health, INSERM U1018, Université Paris-Saclay, France

Hôpital Paul Brousse, Bât 15/16, 16 Avenue Paul Vaillant Couturier

94807 VILLEJUIF CEDEX, France

Telephone: +33 (0)1 45 59 51 52 Fax: +33 (0)1 77 74 74 03

Email: severine.sabia@inserm.fr

[2] Department of Epidemiology and Public Health, University College London, UK

[3] INSERM U1219, University of Bordeaux, Bordeaux, France

Online Only Supplements

Supplementary Methods

Sensitivity analysis for trajectories of physical activity preceding dementia using a case-control approach

Equations

Supplementary Figures

Figure A. Trajectory of global cognitive score in dementia cases and all other participants in the years leading to dementia diagnosis

Figure B. Study flow chart of sample selection, Whitehall II Study

Figure C. Trajectories of physical activity (hours/week) preceding dementia using cubic regression splines

Figure D. Trajectories of physical activity (hours/week) prior to dementia diagnosis using a case-control approach

Figure E. Trajectories of physical activity (hours/week) over 28 years preceding dementia with or without CVD history

Supplementary Tables

Table A. Association of physical activity with performance on the global cognitive z-score at age 50, 60, 70, and 80 years, adjusted for socio-demographic and behavioural factors

Table B. Association of physical activity with the MEMORY z-score at age 50, 60, 70, and 80 years

Table C. Association of physical activity with the REASONING z-score at age 50, 60, 70, and 80 years

Table D. Association of physical activity with the FLUENCY z-score at age 50, 60, 70, and 80 years

Table E. Association of physical activity in 1997/99 with decline over 15 years (1997/99 to 2012/13) in the MEMORY z-score

Table F. Association of physical activity in 1997/99 with decline over 15 years (1997/99 to 2012/13) in the REASONING z-score

Table G. Association of physical activity in 1997/99 with decline over 15 years (1997/99 to 2012/13) in the FLUENCY z-score

Table H. Difference in Physical Activity (hours/week) in the years preceding dementia between dementia cases and others

Table I. Difference in Physical Activity (hours/week) in the years preceding dementia between dementia cases and controls

Table J. Association between physical activity and risk of dementia with history of CVD

Table K. Association between physical activity and risk of dementia without history of CVD

Table L. Association between physical activity and dementia: sensitivity analysis on the influence of dementia misclassification

Supplementary Methods

Sensitivity analysis for trajectories of physical activity preceding dementia using a case-control approach

We used a case-control approach in order to better adjust for confounders, and period effects inherent in the diagnosis of dementia. Index year for dementia cases and their matched controls was year of the first record of dementia diagnosis. Each case was individually matched to 6 controls drawn randomly from the study population using the following criteria: age (5-year age-group at the index year), sex, education, being alive at the index date, and without a diagnosis of dementia at the end of follow-up. The analytic strategy was similar to that in the main analysis, mixed models with a backward scale were used to compare trajectories of physical activity between dementia cases and their matched controls.

Equations

(1) Association of physical activity with cognitive performance at age 50, 60, 70, and 80 years

$$Cognition_{ij} = \beta_0 + \beta_1\, age_{ij} + \beta_2 age_{ij}^2 + \beta_3\, PA_{ij} + \beta_4\, X_i + \beta_5\, Z_{ij} + \beta_6\, C_i + \beta_7\, PA_{ij} age_{ij} + \beta_8\, X_i\, age_{ij} + \beta_9\, PA_{ij} age_{ij}^2 + \beta_{10}\, X_i age_{ij}^2 + U_{0i} + U_{1i} age_{ij} + e_{ij}$$

where $Cognition_{ij}$ is the cognitive score of the ith participant at the jth occasion,

age_{ij} is the age (centred at mean age (65 years) over the follow-up period) of the ith participant at the jth occasion,

PA_{ij} is the physical activity variable for the ith participant at the jth occasion,

C_i is the 5-year birth cohort of ith participant,

X_i is the vector of time independent covariates (sex, education, ethnicity) of ith participant,

Z_{ij} is the vector of time-varying covariates (occupational position, marital status, health behaviours and health-related variables) of ith participant at the jth occasion,

U_{0i} is the random intercept, U_{1i} is the random slope, and e_{ij} is the residual.

(2) Association of physical activity in 1997/99 with cognitive decline over 15 years (1997/99 to 2012/13)

$$Cognition_{ij} = \beta_0 + \beta_1\, time_{ij} + \beta_2 time_{ij}^2 + \beta_3\, PA_i + \beta_4\, age_i + \beta_5\, X_i + \beta_6\, PA_i time_{ij} + \beta_7\, age_i\, time_{ij} + \beta_8\, X_i\, time_{ij} + \beta_9\, age_i\, time_{ij}^2 + U_{0i} + U_{1i} time_{ij} + e_{ij}$$

where $Cognition_{ij}$ is the cognitive score of the ith participant at the jth occasion,

$time_{ij}$ is the delay since 1997/99 for the ith participant at the jth occasion,

PA_i is the physical activity variable of the ith participant in 1997/99,

age_i is the age of the ith participant in 1997/99 (centred at mean age (55 years)),

X_i is the vector of covariates in 1997/99 (sex, education, occupational position, marital status, health behaviours, and health-related variables)

U_{0i} is the random intercept, U_{1i} is the random slope, and e_{ij} is the residual.

(3) Association between physical activity and dementia

$$\lambda\,(a \mid j, z) = \lambda_{0,j}\,(a)\, \exp\,(\beta' Z)$$

where λ is the hazard of dementia at age *a* for an individual with the vector of covariates Z in 1985/88 and $\lambda_{0,j}$ is the baseline hazard for participants born in the jth 5-year birth cohort.

(4) Trajectories of physical activity (hours/week) over 28 years preceding dementia

$$PA_{ij} = \beta_0 + \beta_1\, time_{ij} + \beta_2 time_{ij}^2 + \beta_3 time_{ij}^3 + \beta_4\, Dementia_i + \beta_5\, C_i + \beta_6 age_i + \beta_7\, X_i + \beta_8\, Z_{ij} + \beta_9\, Dementia_i time_{ij} + \beta_{10}\, age_i time_{ij} + \beta_{11}\, X_i\, time_{ij} + \beta_{12}\, Dementia_i time_{ij}^2 + \beta_{13}\, age_i time_{ij}^2 + \beta_{14}\, X_i time_{ij}^2 + \beta_{15}\, Dementia_i time_{ij}^3 + \beta_{16} age_i time_{ij}^3 + \beta_{17}\, X_i\, time_{ij}^3 + U_{0i} + U_{1i} time_{ij} + e_{ij}$$

where PA_{ij} is the physical activity variable for the ith participant at the jth occasion,

$time_{ij}$ is the delay (negative value) between the time when physical activity was measured for the ith participant at the jth occasion and the index date (year=0, corresponding to dementia diagnosis for cases, year of death for those who died, and March 31st 2015 for all others),

$Dementia_i$ is the dementia status at the end of follow-up (0=non-cases, 1=cases),

C_i is the 5-year birth cohort of ith participant,

age_i is the age of the ith participant at the index year (centred at mean age of dementia diagnosis (75 years)),

X_i is the vector of time-independent covariates at Year 0 (sex, education, ethnicity) of the ith participant,

Z_{ij} is the vector of time-varying covariates (occupation position, marital status, and behavioural factors) of ith participant at the jth occasion,

U_{0i} is the random intercept, U_{1i} is the random slope, and e_{ij} is the residual.

Figure A. Trajectory of global cognitive score in dementia cases and all other participants in the years leading to dementia diagnosis.

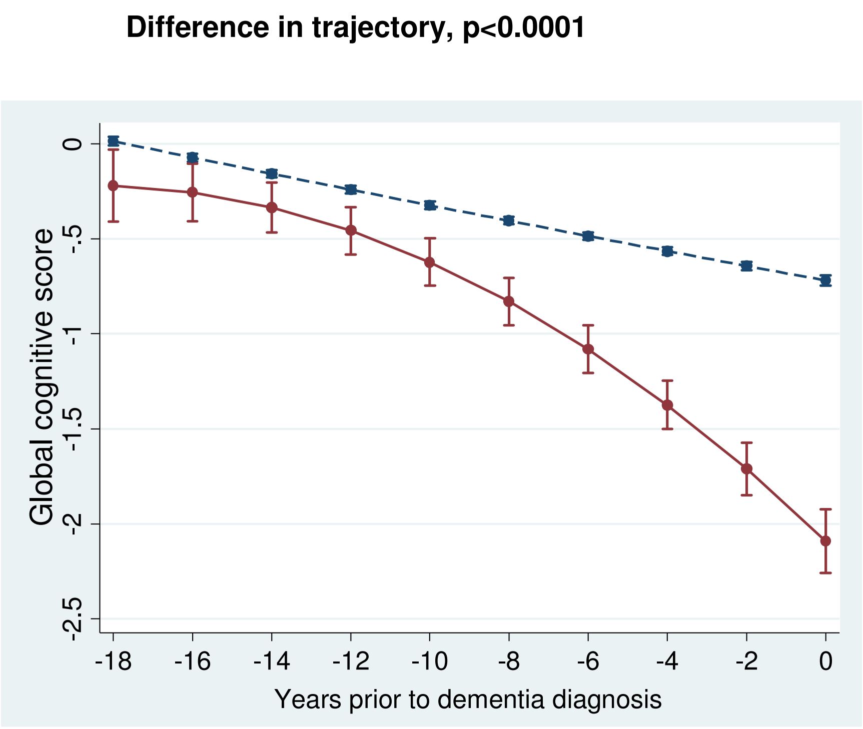

	Number of observations in the analysis				
**Years**	-18 to -16	-16 to -12	-12 to -8	-8 to -4	-4 to 0
**Dementia free (N=7311)**	5192	2489	3714	5938	4777
**Dementia cases (N=195)**	31	81	119	110	89

Legend 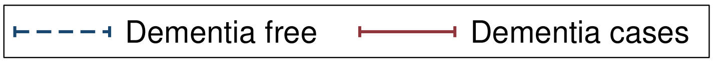

Figure B. Study flow chart of sample selection, Whitehall II Study.

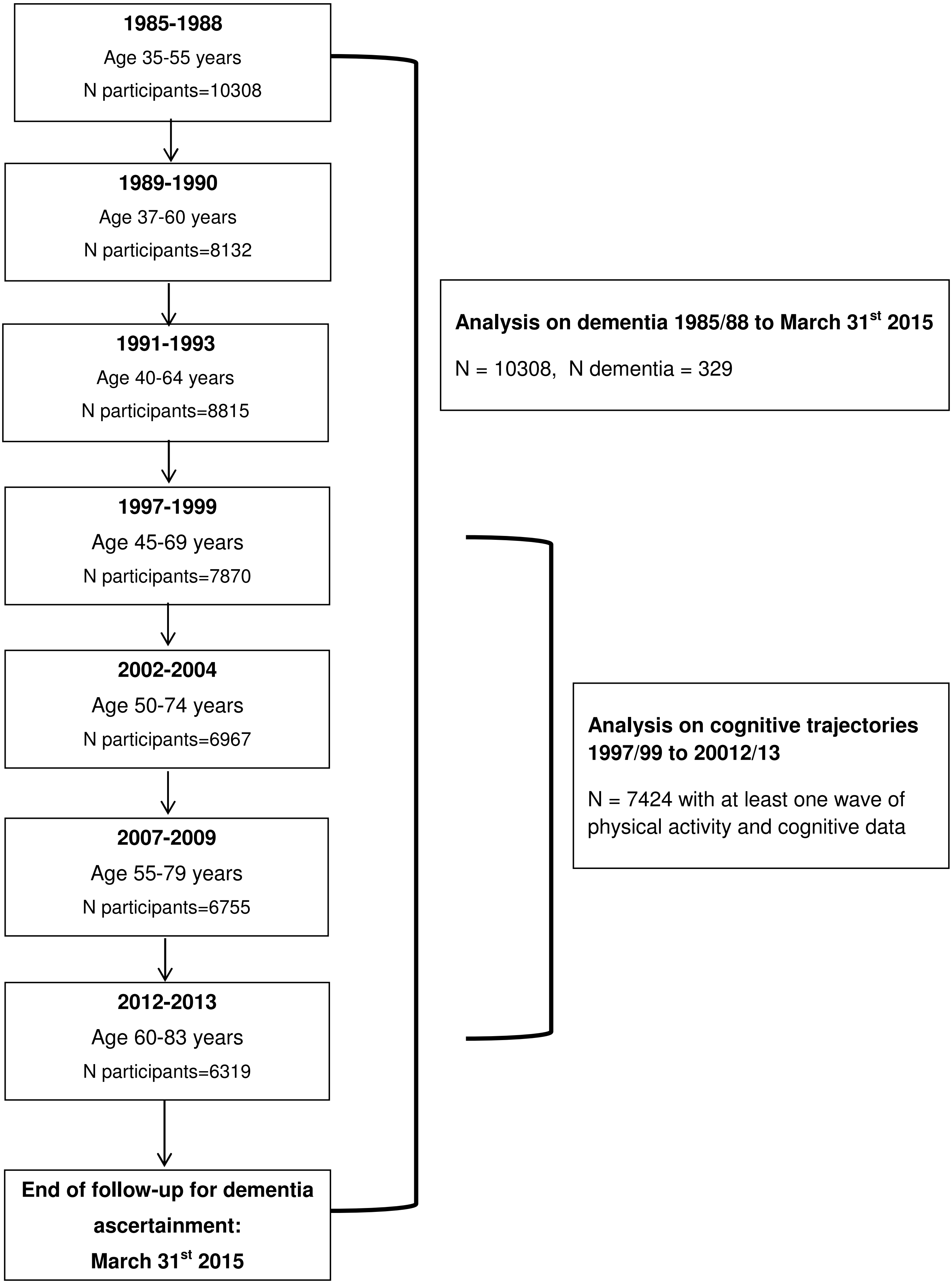

Figure C. Trajectories of physical activity (hours/week) preceding dementia using cubic regression splines

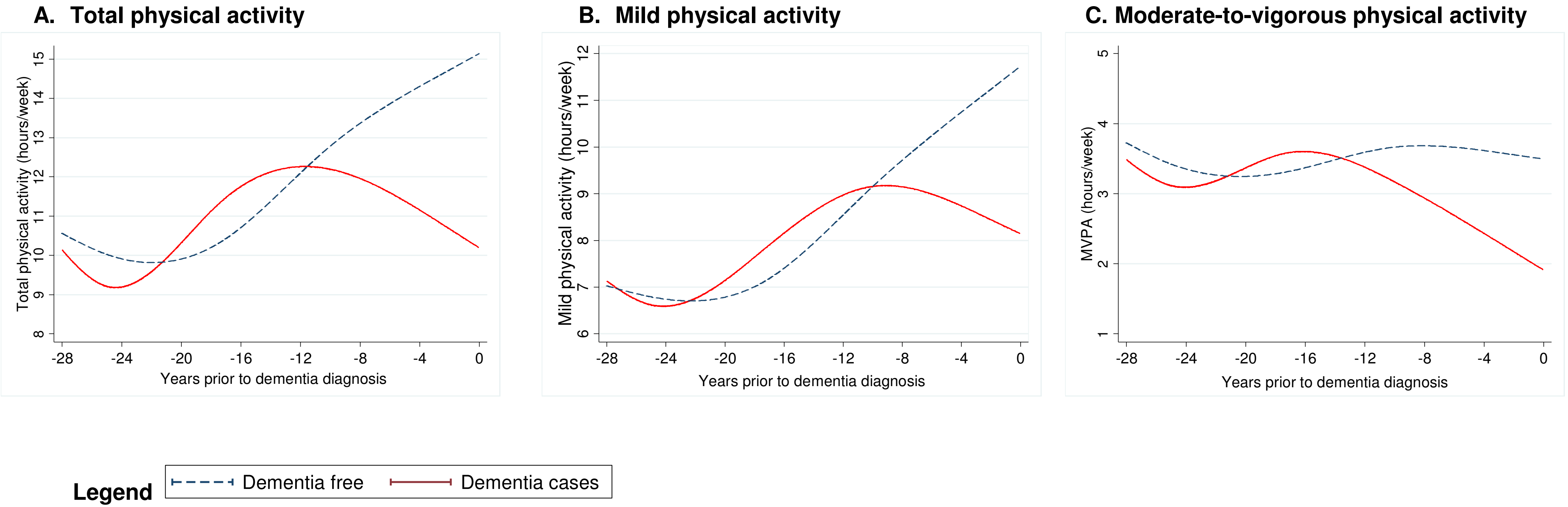

Abbreviation: MVPA, moderate-to-vigorous physical activity.

Figure D. Trajectories of physical activity (hours/week) prior to dementia diagnosis using a case-control approach

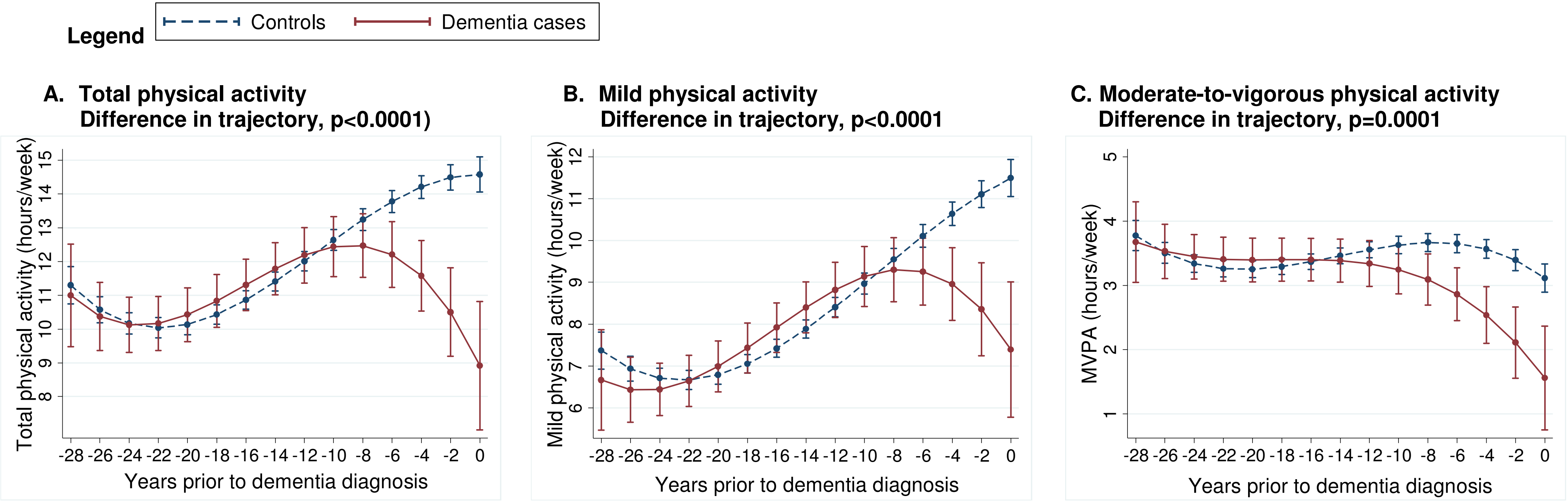

	Number of observations in the analysis																					
**Years**	-28 to -24	-24 to -20	-20 to -16	-16 to -12	-12 to -8	-8 to -4	-4 to 0		-28 to -24	-24 to -20	-20 to -16	-16 to -12	-12 to -8	-8 to -4	-4 to 0		-28 to -24	-24 to -20	-20 to -16	-16 to -12	-12 to -8	-8 to -4	-4 to 0
**Controls (N=1974)**	1822	2260	1347	1186	1313	1148	1509		1822	2260	1347	1186	1313	1148	1509		1822	2260	1347	1186	1313	1148	1509
**Dementia cases (N=329)**	256	298	195	163	168	137	118		256	298	195	163	168	137	118		256	298	195	163	168	137	118

See accompanying table in appendix table S9. Abbreviation: MVPA, moderate-to-vigorous physical activity.

Figure E. Trajectories of physical activity (hours/week) over 28 years preceding dementia with or without CVD history

Legend — Dementia free; Dementia cases without CVD history; Dementia cases with CVD history

A. Total physical activity
Difference in trajectories, p<0.0001

B. Mild physical activity
Difference in trajectory, p<0.0001

C. Moderate-to-vigorous physical activity
Difference in trajectory, p=0.01

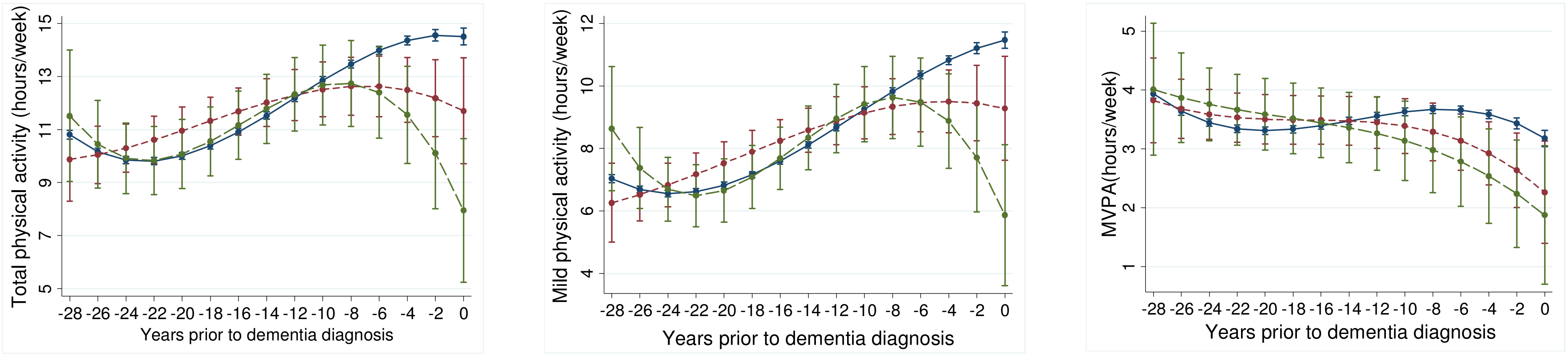

	Number of observations in the analysis																						
**Years**	-28 to -24	-24 to -20	-20 to -16	-16 to -12	-12 to -8	-8 to -4	-4 to 0		-28 to -24	-24 to -20	-20 to -16	-16 to -12	-12 to -8	-8 to -4	-4 to 0		-28 to -24	-24 to -20	-20 to -16	-16 to -12	-12 to -8	-8 to -4	-4 to 0
**Dementia free (N=9979)**	10893	7856	6958	2456	5237	6937	6756		10893	7856	6958	2456	5237	6937	6756		10893	7856	6958	2456	5237	6937	6756
**Dementia cases with CVD history (N=111)**	78	93	59	52	54	39	32		78	93	59	52	54	39	32		78	93	59	52	54	39	32
**Dementia cases without CVD history (N=218)**	178	205	136	111	114	98	86		178	205	136	111	114	98	86		178	205	136	111	114	98	86

Table A. Association of physical activity with performance on the global cognitive z-score at age 50, 60, 70, and 80 years, adjusted for socio-demographic and behavioural factors.

	AGE 50	AGE 60	AGE 70	AGE 80	
	Difference in cognitive function* (95% CI)	Difference in cognitive function* (95% CI)	Difference in cognitive function* (95% CI)	Difference in cognitive function* (95% CI)	*P for interaction with age*
**Total physical activity**					*<0.0001*
**<8 hours/week**	Ref	Ref	Ref	Ref	
**8-12 hours/week**	0.01 (-0.03 to 0.05)	0.00 (-0.03 to 0.02)	0.06 (0.03 to 0.09)	0.20 (0.13 to 0.28)	
**≥12 hours/week**	0.02 (-0.03 to 0.06)	0.01 (-0.01 to 0.03)	0.06 (0.04 to 0.09)	0.18 (0.11 to 0.25)	
**Mild physical activity**					*0.02*
**<5 hours/week**	Ref	Ref	Ref	Ref	
**5-9 hours/week**	0.01 (-0.03 to 0.05)	0.00 (-0.02 to 0.03)	0.05 (0.02 to 0.08)	0.14 (0.06 to 0.22)	
**≥9 hours/week**	0.00 (-0.05 to 0.04)	0.01 (-0.02 to 0.03)	0.05 (0.02 to 0.08)	0.12 (0.04 to 0.19)	
**Moderate-to-vigorous physical activity**					*<0.0001*
**<2 hours/week**	Ref	Ref	Ref	Ref	
**2-4 hours/week**	0.02 (-0.02 to 0.06)	0.02 (-0.01 to 0.04)	0.06 (0.04 to 0.09)	0.16 (0.09 to 0.23)	
**≥4 hours/week**	0.04 (0.01 to 0.09)	0.01 (-0.01 to 0.03)	0.06 (0.03 to 0.08)	0.18 (0.11 to 0.24)	
**Physical activity recommendations†**					*<0.0001*
**MVPA <2.5 hours/week**	Ref	Ref	Ref	Ref	
**MVPA ≥2.5 hours/week**	0.03 (0.01 to 0.07)	0.00 (-0.02 to 0.02)	0.04 (0.02 to 0.06)	0.15 (0.10 to 0.21)	

* Estimated differences (expressed in standard deviations using the distribution of the first wave of cognitive data in 1997/99) in cognitive function at age 50, 60, 70, and 80 years drawn from a single longitudinal model using age at the time scale.

Models are adjusted for ethnicity, education, 5-year birth cohort, and time-dependent occupational position, marital status, smoking status, alcohol consumption, and fruit and vegetable consumption.

†Recommended level corresponds to Moderate-to-Vigorous Physical Activity (MVPA) ≥2.5 hours/week.

Table B. Association of physical activity with the MEMORY z-score at age 50, 60, 70, and 80 years

	AGE 50	AGE 60	AGE 70	AGE 80	
	Difference in cognitive function* (95% CI)	Difference in cognitive function* (95% CI)	Difference in cognitive function* (95% CI)	Difference in cognitive function* (95% CI)	*P for interaction with age*
**Total physical activity**					*0.007*
**<8 hours/week**	Ref	Ref	Ref	Ref	
**8-12 hours/week**	-0.01 (-0.07 to 0.06)	-0.01 (-0.05 to 0.02)	0.05 (0.01 to 0.09)	0.19 (0.07 to 0.31)	
**≥12 hours/week**	0.05 (-0.02 to 0.12)	-0.02 (-0.06 to 0.02)	0.01 (-0.03 to 0.05)	0.15 (0.04 to 0.26)	
**Mild physical activity**					*0.42*
**<5 hours/week**	Ref	Ref	Ref	Ref	
**5-9 hours/week**	0.02 (-0.04 to 0.09)	-0.01 (-0.04 to 0.03)	0.03 (-0.01 to 0.07)	0.13 (0.01 to 0.26)	
**≥9 hours/week**	0.00 (-0.08 to 0.08)	-0.03 (-0.07 to 0.01)	-0.01 (-0.05 to 0.04)	0.08 (-0.04 to 0.20)	
**Moderate-to-vigorous physical activity**					*0.28*
**<2 hours/week**	Ref	Ref	Ref	Ref	
**2-4 hours/week**	0.03 (-0.04 to 0.09)	0.00 (-0.03 to 0.04)	0.02 (-0.02 to 0.06)	0.08 (-0.03 to 0.18)	
**≥4 hours/week**	0.09 (0.02 to 0.16)	0.02 (-0.02 to 0.05)	0.02 (-0.02 to 0.06)	0.10 (0.00 to 0.21)	
**Physical activity recommendations** †					*0.05*
**MVPA <2.5 hours/week**	Ref	Ref	Ref	Ref	
**MVPA ≥2.5 hours/week**	0.06 (0.01 to 0.12)	0.00 (-0.03 to 0.03)	0.02 (-0.02 to 0.05)	0.11 (-0.02 to 0.20)	

* Estimated differences (expressed in standard deviations using the distribution of the first wave of cognitive data in 1997/99) in cognitive function at age 50, 60, 70, and 80 years drawn from a single longitudinal model using age at the time scale.
Models are adjusted for ethnicity, education, 5-year birth cohort, and time-dependent occupational position, marital status, smoking status, alcohol consumption, fruit and vegetable consumption, hypertension, diabetes, BMI, GHQ score, CVD, CVD medication and the SF-36 physical component score.
†Recommended level corresponds to Moderate-to-Vigorous Physical Activity (MVPA) ≥2.5 hours/week.

Table C. Association of physical activity with the REASONING z-score at age 50, 60, 70, and 80 years

	AGE 50	AGE 60	AGE 70	AGE 80	
	**Difference in cognitive function* (95% CI)**	**Difference in cognitive function* (95% CI)**	**Difference in cognitive function* (95% CI)**	**Difference in cognitive function* (95% CI)**	***P for interaction with age***
**Total physical activity**					*0.03*
**<8 hours/week**	Ref	Ref	Ref	Ref	
**8-12 hours/week**	0.01 (-0.03 to 0.04)	0.00 (-0.03 to 0.02)	0.02 (0.00 to 0.05)	0.09 (0.02 to 0.15)	
**≥12 hours/week**	-0.02 (-0.06 to 0.02)	0.01 (-0.01 to 0.03)	0.03 (0.01 to 0.06)	0.06 (0.00 to 0.12)	
**Mild physical activity**					*0.03*
**<5 hours/week**	Ref	Ref	Ref	Ref	
**5-9 hours/week**	0.00 (-0.04 to 0.03)	-0.01 (-0.03 to 0.01)	0.01 (-0.01 to 0.03)	0.07 (-0.01 to 0.14)	
**≥9 hours/week**	-0.02 (-0.06 to 0.02)	0.02 (-0.01 to 0.04)	0.03 (0.01 to 0.06)	0.03 (-0.03 to 0.10)	
**Moderate-to-vigorous physical activity**					*0.001*
**<2 hours/week**	Ref	Ref	Ref	Ref	
**2-4 hours/week**	0.01 (-0.03 to 0.04)	0.02 (0.00 to 0.04)	0.05 (0.03 to 0.07)	0.10 (0.04 to 0.16)	
**≥4 hours/week**	0.00 (-0.03 to 0.04)	-0.02 (-0.04 to 0.00)	0.01 (-0.01 to 0.04)	0.11 (0.05 to 0.16)	
**Physical activity recommendations** †					*0.008*
**MVPA <2.5 hours/week**	Ref	Ref	Ref	Ref	
**MVPA ≥2.5 hours/week**	0.01 (-0.02 to 0.04)	-0.01 (-0.03 to 0.01)	0.01 (-0.01 to 0.03)	0.08 (0.03 to 0.13)	

* Estimated differences (expressed in standard deviations using the distribution of the first wave of cognitive data in 1997/99) in cognitive function at age 50, 60, 70, and 80 years drawn from a single longitudinal model using age at the time scale.
Models are adjusted for ethnicity, education, 5-year birth cohort, and time-dependent occupational position, marital status, smoking status, alcohol consumption, fruit and vegetable consumption, hypertension, diabetes, BMI, GHQ score, CVD, CVD medication and the SF-36 physical component score.
†Recommended level corresponds to Moderate-to-Vigorous Physical Activity (MVPA) ≥2.5 hours/week.

Table D. Association of physical activity with the FLUENCY z-score at age 50, 60, 70, and 80 years

	AGE 50	AGE 60	AGE 70	AGE 80	
	Difference in cognitive function* (95% CI)	Difference in cognitive function* (95% CI)	Difference in cognitive function* (95% CI)	Difference in cognitive function* (95% CI)	*P for interaction with age*
**Total physical activity**					*0.003*
**<8 hours/week**	Ref	Ref	Ref	Ref	
**8-12 hours/week**	0.01 (-0.04 to 0.05)	-0.01 (-0.04 to 0.02)	0.03 (0.00 to 0.06)	0.14 (0.05 to 0.22)	
**≥12 hours/week**	-0.03 (-0.08 to 0.02)	0.00 (-0.03 to 0.03)	0.05 (0.02 to 0.08)	0.12 (0.04 to 0.19)	
**Mild physical activity**					*0.38*
**<5 hours/week**	Ref	Ref	Ref	Ref	
**5-9 hours/week**	-0.01 (-0.06 to 0.04)	0.02 (-0.01 to 0.05)	0.05 (0.02 to 0.08)	0.07 (-0.02 to 0.15)	
**≥9 hours/week**	0.00 (-0.05 to 0.06)	0.02 (-0.01 to 0.05)	0.04 (0.01 to 0.07)	0.06 (-0.02 to 0.14)	
**Moderate-to-vigorous physical activity**					*0.003*
**<2 hours/week**	Ref	Ref	Ref	Ref	
**2-4 hours/week**	0.02 (-0.03 to 0.06)	0.00 (-0.02 to 0.03)	0.04 (0.01 to 0.07)	0.14 (0.06 to 0.21)	
**≥4 hours/week**	0.01 (-0.04 to 0.05)	0.00 (-0.02 to 0.03)	0.05 (0.02 to 0.08)	0.16 (0.07 to 0.22)	
**Physical activity recommendations**†					*0.002*
**MVPA <2.5 hours/week**	Ref	Ref	Ref	Ref	
**MVPA ≥2.5 hours/week**	0.01 (-0.03 to 0.05)	0.00 (-0.02 to 0.02)	0.03 (0.01 to 0.06)	0.12 (0.06 to 0.18)	

* Estimated differences (expressed in standard deviations using the distribution of the first wave of cognitive data in 1997/99) in cognitive function at age 50, 60, 70, and 80 years drawn from a single longitudinal model using age at the time scale.
Models are adjusted for ethnicity, education, 5-year birth cohort, and time-dependent occupational position, marital status, smoking status, alcohol consumption, fruit and vegetable consumption, hypertension, diabetes, BMI, GHQ score, CVD, CVD medication and the SF-36 physical component score.
†Recommended level corresponds to Moderate-to-Vigorous Physical Activity (MVPA) ≥2.5 hours/week.

Table E. Association of physical activity in 1997/99 with decline over 15 years (1997/99 to 2012/13) in the MEMORY z-score.

	Adjusted for age and sex		Adjusted for socio-demographic and behavioural factors*		Fully adjusted†	
	15-y cognitive decline‡ (95% CI)	p§	15-y cognitive decline‡ (95% CI)	p§	15-y cognitive decline‡ (95% CI)	p§
**Total physical activity**						
**<8 hours/week**	-0.48 (-0.53 to -0.43)	Ref	-0.49 (-0.55 to -0.44)	Ref	-0.49 (-0.55 to -0.44)	Ref
**8-12 hours/week**	-0.45 (-0.50 to -0.40)	0.48	-0.46 (-0.51 to -0.40)	0.33	-0.46 (-0.51 to -0.41)	0.24
**≥12 hours/week**	-0.47 (-0.52 to -0.42)	0.88	-0.47 (-0.52 to -0.42)	0.57	-0.47 (-0.52 to -0.42)	0.56
**Mild physical activity**						
**<5 hours/week**	-0.46 (-0.52 to -0.41)	Ref	-0.48 (-0.54 to -0.43)	Ref	-0.48 (-0.54 to -0.42)	Ref
**5-9 hours/week**	-0.49 (-0.53 to -0.44)	0.54	-0.49 (-0.53 to -0.44)	0.89	-0.49 (-0.53 to -0.44)	0.85
**≥9 hours/week**	-0.45 (-0.50 to -0.40)	0.71	-0.45 (-0.50 to -0.40)	0.46	-0.45 (-0.51 to -0.40)	0.50
**Moderate-to-vigorous physical activity**						
**<2 hours/week**	-0.44 (-0.48 to -0.39)	Ref	-0.45 (-0.49 to -0.40)	Ref	-0.44 (-0.49 to -0.40)	Ref
**2-4 hours/week**	-0.48 (-0.54 to -0.42)	0.27	-0.48 (-0.54 to -0.43)	0.30	-0.49 (-0.55 to -0.43)	0.24
**≥4 hours/week**	-0.49 (-0.54 to -0.44)	0.11	-0.50 (-0.55 to -0.45)	0.16	-0.50 (-0.55 to -0.45)	0.14
**Physical activity recommendations#**						
**MVPA <2.5 hours/week**	-0.45 (-0.50 to -0.41)	Ref	-0.46 (-0.51 to -0.42)	Ref	-0.46 (-0.50 to -0.42)	Ref
**MVPA ≥2.5 hours/week**	-0.48 (-0.52 to -0.44)	0.38	-0.48 (-0.53 to -0.44)	0.47	-0.48 (-0.53 to -0.44)	0.45

*Adjusted for age, sex, ethnicity, education, occupational position, marital status, smoking status, alcohol consumption, and fruit and vegetable consumption.
†Additionally adjusted for hypertension, diabetes, BMI, GHQ score, CVD, CVD medication, and physical component score.
‡Estimated cognitive decline over 15 years (expressed in standard deviations using the distribution of the first wave of cognitive data in 1997/99) as a function of physical activity..
§ p for difference in decline in the global cognitive score over 15 years by physical activity groups (drawn from a test of interaction between physical activity categories and time since baseline).
#Recommended level corresponds to Moderate-to-Vigorous Physical Activity (MVPA) ≥2.5 hours/week.

Table F. Association of physical activity in 1997/99 with decline over 15 years (1997/99 to 2012/13) in the REASONING z-score.

	Adjusted for age and sex		Adjusted for socio-demographic and behavioural factors*		Fully adjusted†	
	15-y cognitive decline‡ (95% CI)	p§	15-y cognitive decline‡ (95% CI)	p§	15-y cognitive decline‡ (95% CI)	p§
**Total physical activity**						
**<8 hours/week**	-0.41 (-0.44 to -0.38)	Ref	-0.41 (-0.44 to -0.38)	Ref	-0.41 (-0.44 to -0.38)	Ref
**8-12 hours/week**	-0.41 (-0.44 to -0.39)	0.73	-0.42 (-0.44 to -0.39)	0.76	-0.42 (-0.45 to -0.39)	0.65
**≥12 hours/week**	-0.44 (-0.46 to -0.41)	0.15	-0.44 (-0.46 to -0.41)	0.15	-0.44 (-0.47 to -0.42)	0.10
**Mild physical activity**						
**<5 hours/week**	-0.42 (-0.45 to -0.39)	Ref	-0.43 (-0.46 to -0.40)	Ref	-0.43 (-0.46 to -0.40)	Ref
**5-9 hours/week**	-0.40 (-0.43 to -0.38)	0.27	-0.41 (-0.43 to -0.38)	0.23	-0.41 (-0.43 to -0.38)	0.26
**≥9 hours/week**	-0.44 (-0.47 to -0.41)	0.44	-0.44 (-0.47 to -0.42)	0.47	-0.45 (-0.47 to -0.42)	0.43
**Moderate-to-vigorous physical activity**						
**<2 hours/week**	-0.42 (-0.44 to -0.39)	Ref	-0.42 (-0.44 to -0.39)	Ref	-0.41 (-0.44 to -0.39)	Ref
**2-4 hours/week**	-0.41 (-0.44 to -0.38)	0.64	-0.41 (-0.44 to -0.38)	0.77	-0.41 (-0.44 to -0.38)	0.96
**≥4 hours/week**	-0.44 (-0.46 to -0.41)	0.32	-0.44 (-0.47 to -0.41)	0.28	-0.44 (-0.47 to -0.41)	0.17
**Physical activity recommendations#**						
**MVPA <2.5 hours/week**	-0.42 (-0.44 to -0.39)	Ref	-0.42 (-0.44 to -0.39)	Ref	-0.41 (-0.44 to -0.39)	Ref
**MVPA ≥2.5 hours/week**	-0.43 (-0.45 to -0.40)	0.55	-0.43 (-0.45 to -0.41)	0.46	-0.43 (-0.45 to -0.41)	0.29

*Adjusted for age, sex, ethnicity, education, occupational position, marital status, smoking status, alcohol consumption, and fruit and vegetable consumption.
†Additionally adjusted for hypertension, diabetes, BMI, GHQ score, CVD, CVD medication, and the SF-36 physical component score.
‡Estimated cognitive decline over 15 years (expressed in standard deviations using the distribution of the first wave of cognitive data in 1997/99) as a function of physical activity.
§ p for difference in decline in the global cognitive score over 15 years by physical activity groups (drawn from a test of interaction between physical activity categories and time since baseline).
#Recommended level corresponds to Moderate-to-Vigorous Physical Activity (MVPA) ≥2.5 hours/week.

Table G. Association of physical activity in 1997/99 with decline over 15 years (1997/99 to 2012/13) in the FLUENCY z-score.

	Adjusted for age and sex		Adjusted for socio-demographic and behavioural factors*		Fully adjusted†	
	15-y cognitive decline‡ (95% CI)	p§	15-y cognitive decline‡ (95% CI)	p§	15-y cognitive decline‡ (95% CI)	p§
**Total physical activity**						
**<8 hours/week**	-0.55 (-0.59 to -0.52)	Ref	-0.57 (-0.61 to -0.53)	Ref	-0.57 (-0.61 to -0.53)	Ref
**8-12 hours/week**	-0.59 (-0.63 to -0.56)	0.14	-0.59 (-0.63 to -0.56)	0.44	-0.60 (-0.63 to -0.56)	0.37
**≥12 hours/week**	-0.58 (-0.61 to -0.55)	0.29	-0.59 (-0.62 to -0.55)	0.60	-0.59 (-0.62 to -0.55)	0.54
**Mild physical activity**						
**<5 hours/week**	-0.54 (-0.58 to -0.51)	Ref	-0.56 (-0.60 to -0.52)	Ref	-0.56 (-0.60 to -0.52)	Ref
**5-9 hours/week**	-0.59 (-0.62 to -0.56)	0.06	-0.59 (-0.63 to -0.56)	0.14	-0.60 (-0.63 to -0.56)	0.14
**≥9 hours/week**	-0.58 (-0.62 to -0.55)	0.16	-0.59 (-0.63 to -0.56)	0.18	-0.60 (-0.64 to -0.56)	0.15
**Moderate-to-vigorous physical activity**						
**<2 hours/week**	-0.57 (-0.60 to -0.54)	Ref	-0.59 (-0.62 to -0.55)	Ref	-0.59 (-0.62 to -0.55)	Ref
**2-4 hours/week**	-0.58 (-0.62 to -0.54)	0.70	-0.58 (-0.62 to -0.54)	0.97	-0.59 (-0.63 to -0.55)	0.99
**≥4 hours/week**	-0.58 (-0.62 to -0.55)	0.55	-0.58 (-0.62 to -0.54)	0.85	-0.59 (-0.62 to -0.55)	0.99
**Physical activity recommendations#**						
**MVPA <2.5 hours/week**	-0.57 (-0.61 to -0.54)	Ref	-0.59 (-0.62 to -0.56)	Ref	-0.59 (-0.62 to -0.56)	Ref
**MVPA ≥2.5 hours/week**	-0.58 (-0.61 to -0.55)	0.92	-0.58 (-0.61 to -0.55)	0.61	-0.58 (-0.61 to -0.55)	0.70

*Adjusted for age, sex, ethnicity, education, occupational position, marital status, smoking status, alcohol consumption, and fruit and vegetable consumption.
†Additionally adjusted for hypertension, diabetes, BMI, GHQ score, CVD, CVD medication, and the SF-36 physical component score.
‡Estimated cognitive decline over 15 years (expressed in standard deviations using the distribution of the first wave of cognitive data in 1997/99) as a function of physical activity..
§ p for difference in decline in the global cognitive score over 15 years by physical activity groups (drawn from a test of interaction between physical activity categories and time since baseline).
#Recommended level corresponds to Moderate-to-Vigorous Physical Activity (MVPA) ≥2.5 hours/week.

Table H. Difference in Physical Activity (hours/week) between dementia cases and all others in the years preceding dementia diagnosis

Year	Total physical activity		Mild physical activity		Moderate-to-vigorous physical activity	
	Difference in hours	*p*	Difference in hours	*p*	Difference in hours	*p*
-28	-0.42	0.54	-0.06	0.91	-0.05	0.86
-27	-0.21	0.71	0.00	1.00	0.03	0.91
-26	-0.02	0.97	0.06	0.87	0.10	0.65
-25	0.15	0.72	0.13	0.69	0.15	0.43
-24	0.30	0.45	0.19	0.52	0.19	0.29
-23	0.42	0.27	0.26	0.38	0.22	0.23
-22	0.52	0.17	0.32	0.28	0.23	0.20
-21	0.60	0.12	0.38	0.20	0.23	0.20
-20	0.66	0.09	0.43	0.15	0.22	0.22
-19	0.69	0.07	0.46	0.12	0.20	0.27
-18	0.69	0.07	0.49	0.10	0.17	0.35
-17	0.67	0.08	0.50	0.09	0.13	0.47
-16	0.63	0.10	0.50	0.09	0.08	0.64
-15	0.55	0.15	0.48	0.11	0.03	0.87
-14	0.45	0.24	0.44	0.15	-0.03	0.86
-13	0.32	0.42	0.37	0.24	-0.10	0.59
-12	0.16	0.69	0.28	0.39	-0.17	0.37
-11	-0.02	0.96	0.17	0.62	-0.24	0.21
-10	-0.24	0.59	0.02	0.95	-0.32	0.11
-9	-0.49	0.29	-0.15	0.68	-0.39	0.05
-8	-0.77	0.10	-0.36	0.35	-0.47	0.03
-7	-1.08	0.03	-0.60	0.13	-0.55	0.01
-6	-1.42	0.004	-0.88	0.03	-0.63	0.004
-5	-1.80	0.0004	-1.20	0.004	-0.70	0.002
-4	-2.21	<0.0001	-1.56	0.0003	-0.78	0.0008
-3	-2.65	<0.0001	-1.97	<0.0001	-0.85	0.0005
-2	-3.13	<0.0001	-2.42	<0.0001	-0.91	0.0007
-1	-3.65	<0.0001	-2.91	<0.0001	-0.98	0.002
0	-4.20	<0.0001	-3.46	<0.0001	-1.03	0.005
P for difference in trajectories		*<0.0001*		*<0.0001*		*0.001*

Model adjusted for age (age was entered as time scale in the model), sex, ethnicity, education, and time-dependent occupational position, marital status, smoking status, alcohol consumption, and fruit and vegetable consumption.

Table I. Difference in Physical Activity (hours/week) between dementia cases and controls in the years preceding dementia diagnosis

Year	Total physical activity		Mild physical activity		Moderate-to-vigorous physical activity	
	Difference in hours	*p*	Difference in hours	*p*	Difference in hours	*p*
-28	-0.31	0.71	-0.70	0.28	-0.10	0.76
-27	-0.26	0.69	-0.61	0.24	-0.03	0.91
-26	-0.20	0.71	-0.50	0.23	0.03	0.91
-25	-0.13	0.79	-0.39	0.29	0.07	0.72
-24	-0.04	0.92	-0.27	0.43	0.11	0.57
-23	0.04	0.92	-0.14	0.66	0.13	0.47
-22	0.13	0.76	-0.02	0.94	0.15	0.43
-21	0.21	0.62	0.09	0.78	0.15	0.42
-20	0.29	0.50	0.20	0.55	0.15	0.44
-19	0.35	0.41	0.30	0.37	0.13	0.48
-18	0.40	0.34	0.38	0.24	0.11	0.56
-17	0.44	0.30	0.45	0.16	0.07	0.69
-16	0.44	0.28	0.50	0.12	0.03	0.86
-15	0.43	0.30	0.52	0.11	-0.02	0.92
-14	0.38	0.37	0.51	0.12	-0.08	0.67
-13	0.30	0.49	0.48	0.16	-0.14	0.45
-12	0.17	0.70	0.41	0.25	-0.22	0.26
-11	0.01	0.98	0.31	0.40	-0.30	0.14
-10	-0.20	0.68	0.17	0.66	-0.38	0.06
-9	-0.46	0.36	-0.01	0.97	-0.48	0.02
-8	-0.77	0.13	-0.24	0.56	-0.58	0.008
-7	-1.14	0.03	-0.52	0.22	-0.68	0.002
-6	-1.57	0.003	-0.85	0.05	-0.79	0.0004
-5	-2.07	0.0001	-1.24	0.005	-0.91	0.0001
-4	-2.63	<0.0001	-1.68	0.0003	-1.03	<0.0001
-3	-3.27	<0.0001	-2.18	<0.0001	-1.15	<0.0001
-2	-3.98	<0.0001	-2.75	<0.0001	-1.29	<0.0001
-1	-4.78	<0.0001	-3.39	<0.0001	-1.42	0.0001
0	-5.66	<0.0001	-4.10	<0.0001	-1.56	0.0003
P for difference in trajectories		*<0.0001*		*<0.0001*		*0.0001*

Model adjusted for age (age was entered as time scale in the model), sex, ethnicity, education, and time-dependent occupational position, marital status, smoking status, alcohol consumption, and fruit and vegetable consumption.

Table J. Association between physical activity and risk of dementia with history of CVD.

Physical activity in 1985-88 (mean FU=26.6y)	N cases/ N total	Adjusted for age and sex		Adjusted for socio-demographic and behavioural factors*		Fully adjusted†	
		Hazard ratio (95% CI)	p	Hazard ratio (95% CI)	p	Hazard ratio (95% CI)	p
**Total physical activity**							
**<8 hours**	46/4285	1.00	Ref	1.00	Ref	1.00	Ref
**8-12 hours**	24/2451	0.91 (0.55 to 1.49)	0.70	1.08 (0.65 to 1.79)	0.77	1.15 (0.69 to 1.92)	0.59
**≥12 hours**	41/2572	1.14 (0.75 to 1.74)	0.55	1.26 (0.81 to 1.94)	0.31	1.28 (0.82 to 2.00)	0.27
Per 1 hour/week	*111/10308*	*1.00 (0.98 to 1.02)*	*0.95*	*1.00 (0.98 to 1.02)*	*0.91*	*1.00 (0.98 to 1.02)*	*0.93*
**Mild physical activity**							
**<5 hours**	43/4266	1.00	Ref	1.00	Ref	1.00	Ref
**5-9 hours**	35/3211	1.11 (0.71 to 1.73)	0.66	1.33 (0.84 to 2.10)	0.23	1.39 (0.87 to 2.21)	0.17
**≥9 hours**	33/2831	1.16 (0.73 to 1.84)	0.52	1.16 (0.72 to 1.87)	0.54	1.18 (0.73 to 1.92)	0.50
Per 1 hour/week	*111/10308*	*1.01 (0.99 to 1.03)*	*0.41*	*1.00 (0.98 to 1.02)*	*0.93*	*1.00 (0.98 to 1.02)*	*0.88*
**Moderate-to-vigorous physical activity**							
**<2 hours**	40/3225	1.00	Ref	1.00	Ref	1.00	Ref
**2-4 hours**	32/3078	0.96 (0.60 to 1.53)	0.85	1.42 (0.87 to 2.32)	0.16	1.39 (0.85 to 2.29)	0.19
**≥4 hours**	39/4005	0.96 (0.61 to 1.51)	0.85	1.34 (0.84 to 2.14)	0.22	1.35 (0.84 to 2.16)	0.21
Per 1 hour/week	*111/10308*	*1.00 (0.96 to 1.05)*	*0.97*	*1.01 (0.97 to 1.05)*	*0.54*	*1.01 (0.97 to 1.05)*	*0.52*
**Physical activity recommendations‡**							
**MVPA <2.5 hours/week**	57/4961	1.00	Ref	1.00	Ref	1.00	Ref
**MVPA ≥2.5 hours/week**	54/5347	1.01 (0.69 to 1.47)	0.97	1.30 (0.88 to 1.92)	0.19	1.32 (0.89 to 1.95)	0.17

*Adjusted for age, sex, ethnicity, education, occupational position, marital status, smoking status, alcohol consumption, and fruit and vegetable consumption.
†Additionally adjusted for hypertension, diabetes, BMI, GHQ score, CVD, and CVD medication.
‡Recommended level corresponds to Moderate-to-Vigorous Physical Activity (MVPA) ≥2.5 hours/week.

Table K. Association between physical activity and risk of dementia without history of CVD.

Physical activity in 1985-88 (mean FU=26.6y)	N cases/ N total	Adjusted for age and sex		Adjusted for socio-demographic and behavioural factors*		Fully adjusted†	
		Hazard ratio (95% CI)	p	Hazard ratio (95% CI)	p	Hazard ratio (95% CI)	p
**Total physical activity**							
**<8 hours**	96/4285	1.00	Ref	1.00	Ref	1.00	Ref
**8-12 hours**	47/2451	0.88 (0.62 to 1.25)	0.47	0.89 (0.63 to 1.27)	0.54	0.90 (0.63 to 1.28)	0.55
**≥12 hours**	75/2572	0.98 (0.72 to 1.32)	0.88	0.98 (0.72 to 1.33)	0.89	0.97 (0.71 to 1.32)	0.85
Per 1 hour/week	*218/10308*	*0.99 (0.98 to 1.01)*	*0.39*	*0.99 (0.98 to 1.01)*	*0.32*	*0.99 (0.97 to 1.01)*	*0.28*
**Mild physical activity**							
**<5 hours**	88/4266	1.00	Ref	1.00	Ref	1.00	Ref
**5-9 hours**	73/3211	1.12 (0.82 to 1.53)	0.40	1.14 (0.83 to 1.56)	0.43	1.13 (0.83 to 1.56)	0.44
**≥9 hours**	57/2831	0.92 (0.66 to 1.29)	0.64	0.91 (0.65 to 1.29)	0.61	0.89 (0.63 to 1.27)	0.53
Per 1 hour/week	*218/10308*	*0.99 (0.98 to 1.01)*	*0.62*	*0.99 (0.97 to 1.01)*	*0.56*	*0.99 (0.97 to 1.01)*	*0.50*
**Moderate-to-vigorous physical activity**							
**<2 hours**	83/3225	1.00	Ref	1.00	Ref	1.00	Ref
**2-4 hours**	57/3078	0.84 (0.60 to 1.19)	0.33	0.88 (0.62 to 1.25)	0.49	0.87 (0.61 to 1.24)	0.44
**≥4 hours**	78/4005	0.97 (0.71 to 1.34)	0.86	1.00 (0.72 to 1.38)	0.99	0.99 (0.71 to 1.37)	0.94
Per 1 hour/week	*218/10308*	*0.99 (0.95 to 1.02)*	*0.46*	*0.98 (0.95 to 1.02)*	*0.37*	*0.98 (0.95 to 1.02)*	*0.38*
**Physical activity recommendations ‡**							
**MVPA <2.5 hours/week**	115/4961	1.00	Ref	1.00	Ref	1.00	Ref
**MVPA ≥2.5 hours/week**	103/5347	0.99 (0.76 to 1.30)	0.96	1.01 (0.77 to 1.33)	0.94	0.99 (0.75 to 1.31)	0.93

*Adjusted for age, sex, ethnicity, education, occupational position, marital status, smoking status, alcohol consumption, and fruit and vegetable consumption.
†Additionally adjusted for hypertension, diabetes, BMI, GHQ score, CVD, and CVD medication.
‡Recommended level corresponds to Moderate-to-Vigorous Physical Activity (MVPA) ≥2.5 hours/week.

Table L. Association between physical activity and dementia: sensitivity analysis on the influence of dementia misclassification*

Following vs not following physical activity recommendations	Correlation between classification indices	Odds ratio for dementia (95% CI) Adjusted for age and sex
**Observed data**	NA	1.02 (0.81 to 1.29)
**Simulated data: scenario 1**	0.8	0.82 (0.10 to 1.92)
**Simulated data: scenario 2**	0.6	0.83 (0.08 to 2.60)

*Simulated data were generated assuming sensitivity range between 50% and 80% and specificity range between 97% and 100% with a trapezoidal probability density function. This simulation makes the hypothesis of differential dementia misclassification (i.e. the hypothesis that probability of dementia misclassification depends on physical activity). We simulated two scenarios: first where the correlation between the sensitivity distribution in those following recommendations and the sensitivity distribution in all others was 0.8 and then 0.6 (and similarly for the correlations of the specificity distributions); note that a correlation of 1 corresponds to non-differential misclassification.

Abbreviation: NA, not applicable.
